# Stretchable fiber strain sensors for wearable biomonitoring

**DOI:** 10.1093/nsr/nwae173

**Published:** 2024-05-16

**Authors:** Shumao Xu, Xiao Xiao, Jun Chen

**Affiliations:** Department of Bioengineering, University of California, Los Angeles, USA; Department of Bioengineering, University of California, Los Angeles, USA; Department of Bioengineering, University of California, Los Angeles, USA

Wearable bioelectronics have gained significant attention since they may play a role in transforming current healthcare from a reactive and disease-centric model to a personalized one with a focus on disease prevention and health promotion [1–3]. One key building block in this transformation is the advancement in wearable sensory technology, particularly the design and fabrication of highly sensitive strain sensors that can withstand the mechanical deformations associated with bodily movement [4]. Furthermore, compared with conventional thin film-based wearable bioelectronics, textile bioelectronics offer enhanced healthcare functionality while maintaining superior breathability, wearing comfort, long service life, environmental stability, and mechanical robustness [5]. However, functional fibers, the building blocks of smart textiles, face several developmental challenges. These include the difficulty of loading active materials onto the small and curved surfaces of fibers, the mechanical mismatch between fillers and elastomers which often leads to reduced durability, the need for robust adhesion of sensing materials to prevent wear over time, and the obstacles in generating functional microcracks within conductive materials under strain [1–3].

**Figure 1.**
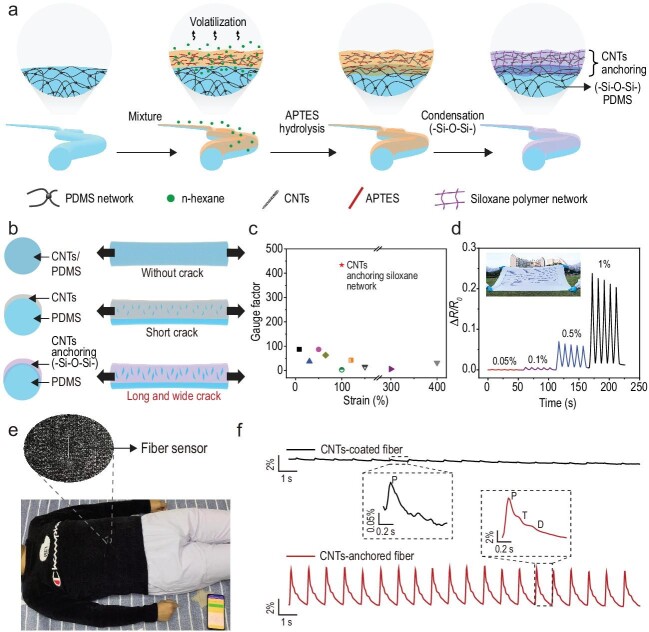
A highly sensitive stretchable fiber strain sensor. (a) Fiber strain sensors fabrication. (b) Schematic of CNTs-filled, CNTs-coated, and CNTs-anchored fiber strain sensor designs, and crack-induced mechanical sensitivity. (c) Gauge factor comparison of CNTs-anchored sensors against other reported sensors across varying strains. (d) Resistance changes under different strain levels, with an inset photo showing integration into commercial fabrics. Wearable CNTs-anchored sensors for (e) breath and (f) pulse monitoring. Reproduced with permission from ref. [6].

A recent study reported by Yu *et al*. [6] develops an ultra-mechanically sensitive stretchable fiber strain sensor using *in-situ* polymerization to anchor and embed conductive sensing materials directly into PDMS substrates, significantly enhancing the mechanical and electrical properties of the fiber strain sensor. This innovative method utilizes the hydrolysis and condensation of a silane coupling agent APTES, 3-aminopropyltriethoxysilane (H_2_NCH_2_CH_2_CH_2_Si(OCH_2_CH_3_)_3_) to create an ultra-mechanically sensitive siloxane polymer network that effectively anchors cabon nanotubes (CNTs) to the PDMS substrate (Fig. 1a). This process begins with a mixture comprising APTES and CNTs in an *n*-hexane solvent sprayed onto a PDMS substrate. The subsequent volatilization of *n*-hexane facilitates the hydrolysis of APTES, R-Si(OCH_2_CH_3_)_3_​ + 3H_2_O → R-Si(OH)_3​_ + 3CH_3_CH_2_OH. Following hydrolysis, these silanol groups undergo a condensation reaction, both among themselves (R-Si(OH)_3_ + R-Si(OH)_3_ → R-Si-O-Si-R + H_2_O) and with any available silanol groups on the PDMS surface (R-Si(OH)_3_ + PDMS-Si-OH→R-Si-O-Si-PDMS + H_2_O), to form robust siloxane (Si-O-Si) bonds. The strong interfacial adhesion resulting from the siloxane bonds facilitates effective stress transfer from the flexible PDMS to the rigid CNTs layer with enhanced mechanical sensitivity. This interaction is crucial when the sensor is stretched, as the mechanical disparity between the CNTs and the PDMS induces microcrack formation within the conductive layer (Fig. 1b), which in turn significantly changes the electrical resistance of the sensor. Traditional methods typically involving dispersing CNTs within the fiber matrix or coating them onto the surface with limited microcrack formation exhibit low gauge factors due to their relatively homogeneous conductive network. However, the CNTs-anchored sensors exhibit a significantly higher gauge factor, demonstrating ∼250-fold increase in sensitivity over traditional models and vastly outperforming others in the literature (Fig. 1c). The formation of microcracks in a siloxane polymer greatly alters the sensor's electrical resistance, thereby boasting an ultra-low strain detection limit of 0.05% and stretchability up to 100%, along with a remarkable gauge factor of ∼430 (Fig. 1c and d). This feature shows great potential in wearable sensors, where monitoring subtle physiological changes such as vibration, pulse, and respiration (Fig. 1e and f) can provide critical data for early diagnosis and health management.

In summary, the work by Yu *et al*. [6] introduces a fiber strain sensor with high sensitivity and excellent durability, addressing several common challenges in wearable bioelectronics related to longevity and reliability. The seamless integration of sensors into textiles opens new avenues for the development of smart clothing capable of real-time monitoring physiological signals like pulse rate and muscle movement in a non-invasive and comfortable manner, constructing textile wearable point-of-care systems to advance the medical fields in the era of the Internet of Things, which could help in preventing diseases, providing treatment, and promoting health anywhere, anytime around the human body.
